# Combination Therapies in Chronic Myeloid Leukemia for Potential Treatment-Free Remission: Focus on Leukemia Stem Cells and Immune Modulation

**DOI:** 10.3389/fonc.2021.643382

**Published:** 2021-05-13

**Authors:** Hui Mu, Xiaojian Zhu, Hui Jia, Lu Zhou, Hong Liu

**Affiliations:** ^1^ Medical School, Nantong University, Nantong, China; ^2^ Department of Hematology, Tongji Hospital, Tongji Medical College, Huazhong University of Science and Technology, Wuhan, China; ^3^ Department of Hematology, Affiliated Hospital of Nantong University, Nantong, China

**Keywords:** chronic myeloid leukemia, tyrosine-kinase inhibitor, drug therapy, immunotherapy, treatment-free remission

## Abstract

Although tyrosine Kinase Inhibitors (TKI) has revolutionized the treatment of chronic myeloid leukemia (CML), patients are not cured with the current therapy modalities. Also, the more recent goal of CML treatment is to induce successful treatment-free remission (TFR) among patients achieving durable deep molecular response (DMR). Together, it is necessary to develop novel, curative treatment strategies. With advancements in understanding the biology of CML, such as dormant Leukemic Stem Cells (LSCs) and impaired immune modulation, a number of agents are now under investigation. This review updates such agents that target LSCs, and together with TKIs, have the potential to eradicate CML. Moreover, we describe the developing immunotherapy for controlling CML.

## Introduction

Chronic myeloid leukemia (CML) is a clonal myeloproliferative disorder, of which the central pathogenic driving event involves the ‘Philadelphia’ chromosomal translocation leading to expression of the BCR-ABL1 fusion oncoprotein. Acting as a constitutively active tyrosine kinase, BCR-ABL1 triggers downstream signaling pathways leading to dysregulated growth and hyperproliferation of leukemic cells (1–7).

The first specific tyrosine kinase inhibitor (TKI) targeting BCR-ABL was imatinib. Its introduction in 2001 completely replaced interferon-alpha (IFN-α) as standard CML treatment, providing high remission rates, fewer side effects and significantly improved patient survival (8–10). Nevertheless, about 20% of CML patients proved resistant and/or intolerant to imatinib (11). This led to the development and introduction of second-generation TKIs with higher selectivity (dasatinib, nilotinib and bosutinib), resulting in more rapid and profound therapeutic milestones (12–20). Thereafter in 2012, the most potent TKI ponatinib was approved for treatment in patients resistant to two or more TKIs, especially those cases developing the common T315I “gatekeeper” mutation that occurs in BCR-ABL in response to TKI therapy (21–23). Asciminib (formerly ABL001), a potent and selective allosteric ABL1 inhibitor, is undergoing clinical development testing in patients with CML and Philadelphia chromosome-positive (Ph^+^) acute lymphoblastic leukemia (ALL). Distinct from all other catalytic-site ABL1 kinase inhibitors (24, 25), the novel compound occupies the myristoyl pocket, a non-ATP-competitive site inducing the BCR-ABL1 kinase protein to adopt an autoinhibited conformation. A preclinical study revealed that ABL001 was active in inhibiting all BCR-ABL1 positive CML cell lines and xenografted mice models derived from either the KCL-22 cell line or primary cells of Ph^+^ ALL patients (26). As expected, the novel compound targets both native and mutated BCR-ABL1, including the T315I mutant. In this respect, it is worth noting that the only clinical agent indicated for T315I mutant cases is ponatinib, which has substantial tolerability issues that limit dosing in CML patients.

Three decades after the introduction of imatinib, the life span of most CML patients has approached to that of the general population (27). Treatment-free remission (TFR) has since then become an additional treatment goal and stop-therapy trials undertaken in recent years have provided proof for the TFR concept (28–34). It is estimated that 30–40% of patients treated with imatinib and 40–50% of patients treated with second-generation TKIs meet the withdrawal criteria (17, 18, 35). To date, the treatment regimens for over 3,000 CML patients reaching a deep molecular response (DMR) has been halted. Thus far, about 50% of patients qualifying for TKI discontinuation have shown sustained TFRs while relapse patients remained sensitive to TKI re-treatment and did not develop BCR-ABL mutations (36). However, it remains poorly understood why these patients relapse within six months of treatment withdrawal (28, 29, 37). There are two main theories for the rapid relapse in these patients, one involving leukemic stem cells (LSCs) and the other being immune surveillance.

**Figure 1:**
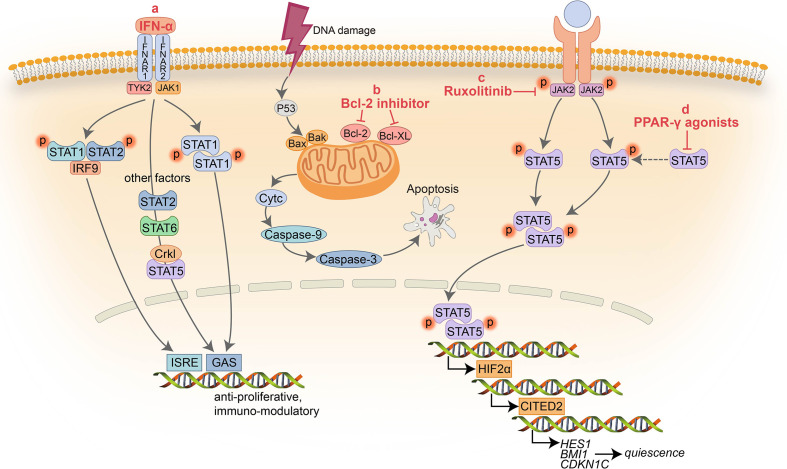
Schematic representation of the signaling pathways targeted by non ABL-directed drugs. **(A)** IFN-α inhibits activation of multiple factors resulting in cell proliferation arrest and immune modulation; **(B)** BCL-2 inhibitors block the pro-survival activity of BCL-2 family members increasing apoptosis; **(C)** Ruxolitinib suppress the activity of JAK2 and downstream factors; **(D)** PPAR-γ agonists serve as a negative transcriptional regulator of STAT5 and its downstream targets HIF2α and CITED2.

Despite their expression of constitutively active BCR-ABL1 kinase, CML LSCs have been shown to be persistent in a quiescent state within the bone marrow niche (38, 39). Although imatinib effectively inhibits BCR-ABL kinase and downstream signaling pathways in stem-like CML cells, it does not induce cell death *in vitro*, suggesting LSCs are resistant to TKI treatment (40). Indeed, in CML patients whose BCR-ABL transcript were undetectable, BCR-ABL^+^ stem cells still could be detected using highly sensitive assays (41–44). Moreover, these and other studies (40, 45, 46) strongly suggest that the survival and proliferation of CML LSCs under TKI treatment occurs through BCR-ABL kinase-independent pathways (**Figure 1**).

Another important factor that likely sustains TFR in CML involves immune surveillance. Suggestively, the elevated natural killer (NK) cells in peripheral blood predict positive clinical outcomes after the discontinuation of imatinib (47), implying that in the context of innate immunity, NK cell-mediated immune surveillance contributes to the control of CML after TKI cessation (48–51). In addition to NK cells, increases in other immune effectors including T and B cell subsets along with concomitantly decreased immune suppressors such as FoxP3^+^ regulatory T cells (Tregs) and myeloid-derived suppressor cells (MDSCs) may also work in concert to mediate a successful TFR (52).

Thus, there is good available evidence to indicate that targeting LSCs or employing immune modulation therapies will bring treatment options closer to TFR for all CML patients. In this review, we focus on updating how combinatorial TKI-based strategies can be used to target LSCs along with strategies for immunological modulation which potentially will induce deeper and faster molecular remission than TKI therapy alone (**Table 1**).

**Table 1:** Clinical studies of TKI-combined therapies and immune strategies for CML.

Therapies	NCT Number	Phase	Patient Characteristics	Status	Primary Objective	Results
**TKI + Interferon**						
Imatnib + interferon	NCT01933906	1	CP-CML (n = 12)	Completed	Safety and tolerability	NDP
	NCT01227356	2	CP-CML (n = 112)	Completed	MMR at 12 months	NDP
	NCT00219739 (SPIRIT)	3	CP-CML (n = 789)	Completed (1, 2)	OS	14% *vs.* 25%: MR4.0 at M12 (IM *vs.* IM + IFN 90 μg/week); 10% *vs.* 28%: MR4.0 (IM *vs.* IM+IFN 45 μg/week)
	NCT00055874	3	CP-CML (n = 1551)	Completed (3, 4)	OS; PFS; molecular response	59% *vs.* 44% *vs.* 46%: MMR at M12 (IM 800 mg/d *vs.* IM 400 mg/d *vs.* IM 400 mg/d + IFN)
Nilotinib + interferon	NCT01220648 (NICOLI)	1	CP-CML (n = 4)	Completed (5)	MTD of IFN	NDP
	NCT01294618 (NILOPEG)	2	CP-CML (n = 41)	Completed (6)	CMR at 12 months	17%: MR 4.5 at M12 24%: grade 3–4 neutropenias 73%: remained on IFN therapy at 1 year
	NCT02001818 (PInNACLe)	2	CP-CML (n = 100)	Recruiting	level of BCR-ABL at 24 months	NDP
	NCT01657604 (TIGER)	3	CP-CML (n = 717)	Active, not recruiting	MMR at 18 months	NDP
	NCT02201459 (PETALs)	3	CP-CML (n = 200)	Unknown	MR4.5 at 12 months	NDP
Dasatinib + interferon	NCT01872442	2	CP-CML (n = NDP)	Completed	MR4.5 at 12 months	NDP
	NCT01725204 (NordCML007)	2	CP-CML (n = 40)	Completed (7)	MMR at 12 months	84%: remained on Peg-IFN at M12 10, 57, 84 and 89%: MMR at M3, M6, M12 and M18 46%: MR4.0 at M12 27%: MR4.5 at M12
Bosutinib + interferon	NCT03831776 (BosuPeg)	2	CP-CML (n = 212)	Recruiting	MR4.0 at 12 months	NDP
						
**TKI + Venetoclax/ABT-199**						
Dasatinib + venetoclax	NCT02689440	2	CP-CML (n = 140)	Recruiting (8, 9)	MMR at M12	81%: MMR; 55%: MR4.0; 49%: MR4.5; 80%: required dose reduction; none: disease progression at M24
Ponatinib + venetoclax	NCT03576547	1/2	Ph^+^ relapsed/refractory ALL/CML (n = 38)	Recruiting	MTD of combination therapy	NDP
	NCT04188405	2	Ph^+^ AML or BP CML (n = 30)	Recruiting	CR or CRi at the end of two cycles of treatment (each cycle is 28 days)	NDP
						
**TKI + Ruxolitinib**						
Nilotinib + ruxolitinib	NCT01702064	1	CML patients with evidence of molecular disease (n = 11)	Completed (10)	MTD of ruxolitinib with nilotinib at M6	1/11: grade 3/4 hypophosphatemia; 36%: grade 1/2 anemia; 4/10: have undetectable BCR-ABL transcripts
	NCT02253277 (CoRNea)	1	CML and Ph^+^ ALL (n = 5)	Completed	DLTs during cycle 1 (up to day 28)	NDP
	NCT01914484	1/2	TKI resistant CML or Ph^+^ ALL (n = 4)	Completed	MTD at M6	NDP
Das/Nil + ruxolitinib	NCT03654768	2	CP-CML (n = 84)	Recruiting	MR4.5 at M12	NDP
either prior TKI + ruxolitinib	NCT03610971	2	CP and previously attempted to discontinue TKI therapy (n = 14)	Recruiting	TFR rate at M12	NDP
						
**TKI + Asciminib**						
Imatinib + asciminib	NCT03578367	2	pre-treated CP-CML (n = 80)	Recruiting	MR4.5 at 48 weeks	NDP
Dsatinib + asciminib	NCT03595917	1	Ph^+^ B-ALL or CML (n = 34)	Recruiting	MTD of ABL001 after 42 Days	NDP
Bosutinib + asciminib	NCT03106779	3	CML-CP previously treated with two or more TKIs (n = 233)	Active, not recruiting	MMR rate of ABL001 versus bosutinib at 24 weeks	NDP
IM/NIL/DAS + asciminib	NCT02081378	1	CML or Ph^+^ ALL relapsed/refractory/intolerant to TKIs (n = 330)	Recruiting	DLTs during the first cycle of treatment (first cycle is 28 days)	48%: MMR by 12 months; MMR was achieved/maintained by 12 months in five patients (28%) with a T315I mutation
	NCT03906292 (CMLXI)	2	newly diagnosed CML (n = 120)	Recruiting	MR4.0 at M12	NDP
						
**TKI + Pioglitazone**	NCT02852486 (EDI-PIO)	2	CML with DMR (n = 31)	Active, not recruiting (11)	TFR time after IM discontinuation	NDP
	NCT02889003 (PIO2STOP)	2	CML (n = 26)	Recruiting	Treatment-related adverse events and treatment-free survival up to 24 months	NDP
	NCT02767063 (ACTIW)	1	CP-CML in CCR (n = 100)	Recruiting	DMR at M12	NDP
						
**TKI + Vaccines**						
BCR-ABL1 as a specific antigen						
e13a2	NCT00466726 (CML0206)	2	CML (n = 57)	Completed (12)	Reduction by at Least 50% of peripheral blood BCR-ABL/ABL ratio at 6 and 9 months	9%: a mild fever; 67%:CD4^+^ T cell proliferation; 51%: a reduction of ≥50% of pre-vaccine BCR-ABL/ABL values after nine vaccinations; 48%: the reduction was confirmed after 10 vaccinations
	NCT00004052	2	CML (n = 24)	Completed	Safety and immunogenicity	NDP
e13a2, e14a2	NCT00428077	2	CP (n = 4)	Terminated	BCR-ABL transcripts in PB every 3 months for 1 year	1/4: grade 3 acute gastroenteritis; 1/4: grade 2 cataracts
	NCT00267085	2	CML in remission but with MRD (n = 10)	Completed	One Log Decrease in BCR-ABL at M12	1/10: nausea; 1/10: neck pain; 1/10: back pain
LAAs						
WT1	NCT00004918	1/2	CML, AML or MDS (n = 69)	Completed	Adverse event DTOX up to 8 years	NDP
						
**TKI + ICB**						
Nivolumab	NCT01822509	1	Relapsed hematologic malignancies including CML (n = 71)	Active, not recruiting (13)	MTD of nivolumab at 12 weeks	32% (8/25): ORR 23%: 1-year PFS 56%: OS
	NCT02011945	1	CML (n = 35)	Completed	DLT of combination therapy during the first 6 weeks	NDP
Avelumab	NCT02767063 (ACTIW)	1/2	CP-CML in CCR (n = 100)	Recruiting	DMR at M12	NDP

TKI, tyrosine kinase inhibitor; IM, imatinib; NIL, nilotinib; DAS, dasatinib; LAAs, leukemia associated antigens; ICB, immune-checkpoint blockade; OS, overall survival; PFS, Progression-free survival; NDP, No Data Posted; MTD, maximum tolerated dose; IFN, interferon; CMR, complete molecular remissions; DMR, deep molecular response; ALL, Acute lymphoblastic leukemia; AML. Acute Myeloid Leukemia; MDS, Myelodysplastic Syndrome; BP, blast phase; CR, complete remission; Cri, CR with incomplete count recovery; DLTs, dose limiting toxicities; PB, Peripheral blood; DTOX, death or autoimmune toxicity or vascular toxicity at any time; ORR, overall response rate; PFS, progression-free survival.

## TKIs + Interferon-α (IFN-α)

IFN-α therapy was first reported in the 1980s to show hematological responses in CML (53, 54). Compared with conventional chemotherapy, IFN-α monotherapy proved to delay disease progression and prolong overall survival. Thereafter in the 1990s, IFN-α became the standard therapy for CML patients who were not suited for bone marrow transplantation (55–60). However, the emergence of imatinib largely displaced IFN-α in clinical practice, and was only used during pregnancy or for patients with TKI intolerance (61–63). Nevertheless, in the current era IFN-α is making a comeback in CML therapy due to its unique activity and immunological effects against CML LSCs.

Notably, patients previously treated with IFN-α demonstrate an increased likelihood of TFR compared to TKI monotherapy (64). Furthermore, IFN-α together with TKI therapy showed positive effects through achieving deeper molecular responses and eliminating the T315I mutation (65). *In vitro* studies have demonstrated that IFN-α exerts its anti-leukemic effect mainly by directly inhibiting the proliferation of CML progenitor cells. Here, IFN-α efficiently target key regulators of cell cycle progression such as cdc25A, thus blocking or lengthening the cell cycle and causing cells to differentiate or undergo apoptosis (66, 67). Towards the latter, IFN-α activates apoptosis by inducing expression of pro-apoptotic proteins such as Fas/CD95, so as to increase cell sensitivity to Fas ligand (68, 69). It was further observed in a mouse model that IFN-α efficiently reactivates LSC entry into the cell cycle (70). Together these reports suggest that IFN-α acts to sensitize and help eliminate LSCs, thereby providing a rational basis for the association between IFN-α treatment and improvements in treatment outcomes.

In addition to these direct actions against LSCs, it was evident from earlier reports in the 1990s that IFN-α treatment modulates immunity through pleiotropic effects. In particular, IFN-α strengthens anti-tumor immunity by activating autoimmune cells including NK cells, B and T lymphocytes and antigen-presenting cells (APC) (71). *In vitro* modeling of autologous and non-autologous NK : CML cell-interactions demonstrated that IFN-α treatment stimulated NK cytolytic activity (72, 73). Moreover, recent studies suggest IFN-α as a treatment strategy to boost immune surveillance and potentially eradicate LSCs if combined with careful monitoring of immunosuppressive cells (74). Bringing together the aforementioned concepts, several clinical CML studies combining TKI with IFN-α have been reported and representative studies among these are summarized in **Table 2**.

**Table 2:** Four classical clinical trials of TKI+IFN combination therapy for CML patients.

Trial	Treatment regimen (median adminstrated dose)	Number enrolled	Disease stage; duration from diagnosis	Median follow up; months	CHR%(m)	CCyR% (m)						MMR% (m)						DMR% (m)					Undetectable BCR-ABL1 % (m)				
					3	6	12	18	24	36	48	6	12	18	24	36	48	6	12	18	24	36	6	12	24	36	48
Italian GIMEMA	IM (400mg/d) +peg IFN-α2b (33μg/w)	76	CP; ≤6 months	60m		60	70		81	87	82	58	67		65	65	65						13	15	12	19	19
	IM (400mg/d)	419		43m		42	68		80	82	82	34	47		62	58	57						2	5	18	24	18
French PIRIT	IM (400mg/d)	159	CP; ≤3 months	44m	89	50	58						38	42	43				14	18	21				9		
	IM (600mg/d)	160		46m	89	69	65						49	50	53				17	22	26				8		
	IM (400mg/d) +cytosine arabinoside(14mg/m*2/d)	158		43m	95	59	70						46	53	54				15	19	26				8		
	IM (400mg/d) +Peg IFN-a2a (54 μg/w)	159		44m	91	57	66						57	62	64				30	35	38				16		
German CML-Study IV	IM (400mg/d)	325	CP; ≤ 16d	43m		21	49	66	74			9	31	50	63	79		3	8	21	31	46					
	IM (800mg/d,actually 628mg/d)	338		28m		32	63	75	82			18	55	68	76	82		4	20	33	43	57					
	IM (400mg/d) +IFN-α(1.5m.U-3m.U *3 times/week)	351		48m		20	50	69	77			8	35	54	63	71		2	12	24	30	41					
Nordic roup study	IM (400mg/d) +peg IFN-α2b (42μg/week)	56	CP; <3 months	52w			91						82														
	IM (400mg/d)	56		52w			84						54														

TKI, tyrosine kinase inhibitor; IFN, interferon; CML, chronic myeloid leukemia; IM, imatinib; CP, chronic phase; CHR, complete hematologic response; CCyR, complete cytogenetic response e.g.Ph+ 0%; MMR, major molecular response e.g. BCR-ABL1(IS)≤0.1%; DMR, deep molecular response e.g.BCR-ABL1(IS)≤0.01%.

Interestingly, one critical observation made in the earlier studies concerned dose-dependent effects where high dose interferon limited co-treatment efficacy (75). Consequently, IFN-α dose reductions markedly improved combination therapy with imatinib, showing better tolerance and higher molecular remission rates across multiple studies. The Italian GIMEMA working group enrolled new-diagnosed CML patients to explore the effects of imatinib (n = 76) and imatinib plus pegylated IFN-a (n = 419). At 6 months, both the CCyR rate (60% *vs.* 42%) and MMR rate (58% *vs.* 34%) were higher in the IM + IFN-α group than in the imatinib group. However, a high proportion of the Peg-IFN-α study arm patients permanently discontinued treatment in the first year (45/76; 59%), increasing a further 28% at 2 years (75), ultimately accounting for no differences in long-term remissions (76).

Another phase II study (77) by the Nordic group (n = 112) confirmed the long-term effects of combining peg-IFNα with imatinib. MMR rates in the combination arm were significantly higher compared with imatinib monotherapy (82% *vs.* 54%, respectively) at 12 months. Nonetheless, despite 34 (61%) of patients stopping peg-IFNα treatment due to adverse events, those patients who continued combination therapy for more than one year, reached 91% MMR compared to 58% for imatinib monotherapy. In accordance with these findings, the large French SPIRIT trial observed faster and better molecular responses in the interferon-imatinib arm than for treatment with imatinib alone (n = 159 in each arm). The rate of major and superior (>MR4.0) molecular responses were higher at 12, 18 and 24 months in the co-treatment group with the longer durations of combination therapy associated with better molecular responses. The majority of patients receiving peg-IFN for more than 12 months reached MMR (82%) and 49% achieved MR4 after 2 years in contrast to patients treated only with imatinib (MMR, 43%; MR4, 21%) (78). However, the findings of another large clinical study did not concur with the SPIRIT trial, rather proposing benefits for using higher doses of imatinib.

The German CML-Study IV randomized 1,014 patients to one of the three cohorts: 1) monotherapy imatinib 400 mg QD; 2) imatinib 400 mg QD plus IFNα (1.5–3 mill. U three times per week); or 3) imatinib 800 mg QD. Overall findings demonstrated that the imatinib plus IFNα arm was almost equivalent to standard-dose (400 mg) imatinib whereas high-dose imatinib (800 mg) showed superior results. At 12 months, higher MMR rates were observed in the modified high-dose imatinib arm (800 mg early stage and 600 mg maintenance QD dose) compared with the imatinib 400 mg plus IFN-α arm (55 *vs* 35%, respectively). Similar benefits occurred in CCyR rates over the first 24 months (82 *vs* 77%, respectively) along with the International Scale (IS) molecular responses measuring levels of *BCR-ABL* transcript at 1, 0.1 and 0.01% (79). Discrepancies between the SPIRIT and Study IV results could not be attributed to patient demographics, rather the reason proposed to account for the discordant therapeutic outcomes was the longer circulating half-life of the pegylated IFNα used in the SPIRIT trial (80). Notwithstanding this point, the tolerability-adapted strategy applied in the high-dose imatinib arm of the German CML-Study IV correlated with the superior remission rates.

In summary, the combined treatment of imatinib and interferon improves the depth and speed of disease remission, but toxicity concerns are also significant, which lead to high rates of interferon-treatment termination.

As mentioned in the *Introduction*, the manifestation of imatinib resistance has driven the development of second generation (2G) TKIs with superior activity compared to imatinib in both facilitating molecular remission and preventing disease progression (16, 19, 81). A natural progression of the findings with interferon-imatinib combination therapies saw peg-IFNα added with dasatinib or nilotinib therapies. Our group provided an early exemplary case where a CML patient harboring the T315I and E255V *BCR-ABL1* mutations achieved successful DMR using dasatinib combined with IFN-α (82). Thereafter, this concept has been applied in a diverse range of clinical trials in CML patients. During CML trial NCT01725204, dasatinib (100 mg pd) was combined with pegylated interferon-α2b (peg IFN) at three months (M3) at an initial dose of 15 μg/week, later increasing to 25 μg/week from M6 to M15. A steep rise in response rates was noted after introduction of peg IFN with progressively increasing MMR achieved over time (M3, 10%; M6, 57%; M12, 84% and M18, 89%, respectively). MR4 and MR4.5 were also achieved by a respective 46 and 27% of patients at M12 (83). The recently completed NCT01872442 trial dealt with effectiveness and safety of dasatinib in combination with low dosage of Peg-IFNα2b as first line treatment for newly diagnosed chronic phase (CP) CML patients.

Synergistic effects of Peg-IFNα2a and nilotinib has also been demonstrated in French NiloPeg study. Forty-one newly diagnosed CML patients received the combination therapy with 45 μg peg IFN-2a weekly (90 μg weekly in the first month) and 600 mg nilotinib daily, resulting in MR4.5 in seven (17%) patients at 12 months. Despite hematological and hepatic adverse events, most patients (73%) remained on IFN therapy for more than one year (84). The follow-on French PETALS study (NCT02201459) is comparing nilotinib 600 mg BID against nilotinib 600 mg BID plus peg-IFN2a at increased doses for 24 months. An interim analysis, which evaluated cumulative rates of MR4.5 12 months after nilotinib initiation in 200 newly diagnosed CP-CML patients, showed statistically significant DMR rates in favor of the combination treatment arm at 12 months (85). Similarly, interim results of the PINNACLE study also suggested that combination therapy with nilotinib plus Peg-IFNa2b results in favorable rates of molecular responses compared with nilotinib as monotherapy (86). Finally, the currently ongoing TIGER (CML V)-Study (NCT01657604) aims to investigate if nilotinib 600 mg BID monotherapy would be improved using low dose Peg-IFNα2b (30–50 μg/week) as an inducer of immunosurveillance. According to a per protocol interim analysis, Peg-IFN, when added upfront to nilotinib further increases the rates of MR4.0 and MR4.5, which may translate into higher rates of TFR (87). For NCT02001818, long-term data such as survival length of minimal disease and time to disease progression will be assessed among patients injected with peg-IFN for 2 years.

The combination of bosutinib with interferon is being investigated in the BosuPeg trial, but no results have been published (NCT03831776). Of note, a novel generation of mono-pegylated interferon, ro-peg-IFNa2b, is used in this study. Its half-life is longer, which allows to be administrated once every 14 days (88).

## TKI (NIL/IM) + BCL-2 Inhibitors (Venetoclax/ABT-199)

BCL-2 protein functions as the central abrogator of apoptotic signals by preserving the integrity of the mitochondrial outer membrane (MOM) (89), acting directly to inhibit the efflux of cytochrome-*c* and the activation of caspases (90). In contrast, activation of the pro-apoptotic BH3-only molecules (BH3s) BAX and BAK changes their conformation to mediate cytochrome-*c* release and initiate the mitochondrial apoptotic cascade (91). Inspired by the role of the BH3 subgroup proteins in programmed cell death, a variety of BH3 mimetics appeared as anti-cancer compounds. These agents interact with the anti-apoptotic proteins (BCL-2, BCL-X_L_, and BCL-w) and inhibit their function.

BCL-2 protein is important for the survival of leukemia cells and LSCs (92–94). The expression of BCL-2 in CML is higher than that in normal hematopoietic stem cells (HSCs), and it is further increased if patients progress to blast crisis (BC) CML (95). Notably, BCR-ABL supports CML cell survival by partially upregulating anti-apoptotic BCL-2 proteins (96–98). Emerging data now suggests that the combining TKIs with either BCL-2/BCL-X_L_ or pan-BCL-2 inhibitors can selectively enhance cytotoxicity and thus eradicate CML stem/progenitor cells (95, 99, 100).

The orally bioavailable BH3 mimetic ABT-263 (navitoclax), a potent BCL-2/BCL-X_L_ inhibitor (101), has now entered the clinical trial phase for hematologic malignancies. However, although it displays activity against CML progenitors, the prospects for ABT-263 as a therapeutic agent are hampered by its inhibitory effect on BCL-X_L_, which is critical for platelet survival (102–104). Alternatively, minor structural modifications of ABT-263 have resulted in ABT-199 (venetoclax), a BCL-2 inhibitor sparing BCL-X_L_ but with strong anti-tumor activity (105). A number of preclinical studies have now shown that ABT-199 displays efficacy against various hematological malignancies (106–109). For instance, ABT-199 is now indicated for recurrent or refractory chronic lymphoid leukemia (CLL) with 17p deletion and furthermore, has entered clinical trials for lymphoma and multiple myeloma.

Regarding CML, a 2014 study claimed that ABT-199 significantly enhanced imatinib-mediated apoptosis of early and late CML progenitors at concentrations that avoid hematologic toxicities (110). A later report investigated the effect of combined strategies concomitantly targeting BCR-ABL kinase and BCL-2 with nilotinib and ABT-199 on LSCs *in vivo* and *in vitro* (111). Consistent with previous reports (96, 98), BCL-2 protein expression was markedly increased in BCR-ABL transgenic mice, suggesting that BCL-2 plays a key role in the survival of CML cells. Correspondingly, the combination treatment was more effective than single drugs. Although ABT-199 could induce apoptosis alone, its combination with nilotinib demonstrated enhanced efficacy against both bulk and CD34^+^ cells obtained from patients irrespective of their clinical response to TKIs. As a possible mechanism, they found combination treatment greatly decreased downstream BCR-ABL signaling, especially effected on phosphorylated CRKL (p-CRKL) and MCL-1 expression. Another important finding concerned the protective effect of MSCs in the bone marrow niche which is known to protect acute leukemia cells from therapy. Co-culture of MSCs with BC-CML cells resulted in the increased the expression of the anti-apoptotic proteins BCL-2, BCL-XL and MCL-1, suggesting MSCs play similar conspirator role in CML. Fortunately, combination therapy proved highly synergistic in inducing apoptosis of proliferative and quiescent CML cells, even when co-cultured with MSCs.

Altogether, these results highlight that mechanism-guided double blockade of BCL-2 and tyrosine kinase may cure CP-CML and potentially also BC-CML patients, making this approach worthy of further clinical testing.

As of 2016, a phase 2 clinical trial sponsored by the M.D. Anderson Cancer Center (MDACC) is currently underway that combines 50 mg dasatinib daily with venetoclax for newly diagnosed CP-CML (NCT02689440). As designed, 140 patients enrolled are divided into two arms: I 12 months of treatment with dasatinib 50 mg orally daily; II starting combined treatment with dasatinib 50 mg daily with venetoclax 200 mg daily after 3 months of dasatinib therapy in the absence of disease progression or unacceptable toxicity. Early results from this trial have reported the safety and efficacy of the lower dose of dasatinib monotherapy (50 mg daily) in the treatment of 75 newly diagnosed CML-CP patients (112). The rates of CCyR at 6 and 12 months were 86 and 88%, respectively while at 12 months, 79, 71, and 46% of the well tolerated patients had achieved MMR, MR4.0, and MR4.5, respectively. After a minimum follow-up of 12 months, the updated cumulative rates for MMR, MR4.0 and MR4.5 were 81, 55, and 49%, respectively, which continued to support 50 mg of dasatinib daily as an effective and safe dose for early CML-CP. Two further trials of the combination of ponatinib and venetoclax are also under way (NCT04188405 and NCT03576547).

## TKI + JAK2 Inhibitor (Ruxolitinib)

The janus kinase/signal transducers and activators of transcription (JAK/STAT) pathway are well known as the main signaling axis of many important cytokines (113). During normal hematopoiesis, cytokines bind to and activate their corresponding receptors resulting in intracellular JAK2 phosphorylation of the STAT5 transcription factor which translocates to the cell nucleus to regulate gene transcription (114).

In CML, constitutive activation of the JAK2/STAT5 axis provides possible oncogenic signals for BCR-ABL expressing cells (6, 115). The central role of JAK2/BCR-ABL protein complex was demonstrated to stabilize BCR-ABL kinase activity and interrupting this complex increased the clearance of BCR-ABL^+^ cells, including CML stem/progenitor cells (116–118). Similarly, the consensus has been reached that high levels of STAT5 have a protective effect on BCR-ABL^+^ cells even those treated with TKIs (119). Indeed, targeting STAT5 activity can specifically increase the elimination of BCR-ABL^+^ cells, both primary CML cells in BM as well as CML cells resistant to TKI (120). Therefore, pharmacologic intervention of the JAK2/STAT5 pathway appears a promising strategy for treating CML.

Targeting STAT5 *per se* is problematic since it is not a kinase and lacks enzymatic domains necessary for conventional inhibitor design approaches. Therefore, targeting JAK2 is considered as an alternative method for interfering with the function of STAT5. Notably, JAK2 may also directly interact with other targets of interest other than STAT5. For example, JAK2 directly phosphorylates the key tyrosine 177 residue of BCR-ABL (118), leading to activation of the RAS/MAP kinase pathway (121, 122). The latter is particularly relevant given its activation of MYC (123) and β-catenin (124), both known to play central roles in CML LSC self-renewal (125, 126). Thus, there may be multiple therapeutic benefits realized by targeting JAK2 in CML.

Among numerous small-molecule JAK2 inhibitors emerging in clinical development, ruxolitinib (RUX) stands out as an effective oral JAK1/2 inhibitor (127). This agent has already been licensed for treating primary myelofibrosis based on phase 3 clinical trial data (128, 129). For application to CML, the preclinical evidence supporting the synergy between ruxolitinib at clinically achievable concentrations and nilotinib is very promising (130, 131). Targeting the JAK2/STAT5 pathway showed effective reductions in viability, colony output, and proliferation of CML CD34^+^ cells *in vitro* as well as the engraftment of CML CD34^+^ cells *in vivo*. In this study, the combined treatment further downregulated the levels of *p*-JAK2 and *p*-STAT5 phosphoproteins along with genes regulated by STAT5 including the cell cycle promoters *Cyclin D1*, *D2*, *D3* and the anti-apoptotic gene *BCL-XL*. Interestingly, ruxolitinib was modestly effective as a single agent, but showed highly significant effects when used in combination with nilotinib. The exact mode of action was unclear, but the findings suggested the effects of JAK2 are dispensable when BCR-ABL is fully activated, but under nilotinib inhibition, the role of JAK2 became particularly prominent. Thus, the importance of JAK2 inhibition only became meaningful after inhibiting BCR-ABL, which helps propose the necessity for continuing TKI in conjunction with novel therapeutic agents for CML LSC eradication. However, the main problem when applying JAK2 inhibitors to CML is their potential toxicity to normal bone marrow (132). Nevertheless, the above demonstrates that the conjunction of ruxolitinib and nilotinib can selectively eradicate CML *in vivo* and *in vitro* by tuning the appropriate concentration of ruxolitinib.

Other unanticipated actions of ruxolitinib against CML LSCs also involve a potential immune mechanism involving the regulation of MHC molecules (133). Malignant cells can lose MHC expression to evade immune-mediated clearance by tumor-specific T cells. Indeed, gene expression analyses show MHC-II and its master regulator CIITA are significantly down-regulated in CML stem/progenitor cells. The expression of MHC-II or CTIIA in CML cells was not affected by TKI treatment indicating their expression was independent of BCR-ABL. In contrast, ruxolitinib enhanced the expression of MHC-II in CML stem/progenitor cells, proposing this may help unmask their invisibility to the immune system.

A phase 1 clinical trial investigating the safety and tolerability of ruxolitinib when combined with nilotinib in the treatment of CP-CML patients and has provided some support for the perspectives outlined above. Kendra and colleagues designed the study to determine the maximum tolerated dose (MTD) of ruxolitinib and establish a toxicity profile. All 11 patients remained on their usual doses of nilotinib (300 mg bid versus 400 mg bid, n = 8 versus n = 3) prior to this trial and ruxolitinib was added at 5, 10, and 15 mg bid in three dose cohorts respectively. After 6 months of combination therapy, ruxolitinib was discontinued and patients continued to receive nilotinib treatment without dose adjustment. All five patients who took ruxolitinib 15 mg twice daily were also given nilotinib 300 mg bid. The most common adverse reactions at all dose levels were mild hyperbilirubinemia (64%) and elevated alanine aminotransferase (45%). One patient developed grade 3 hypophosphatemia, which was successfully treated by oral potassium phosphate. Overall, no dose-limiting toxicities was observed in patients with the combination intervention, and no dose adjustment was required based on the treatment-emergent adverse events (134). In general support of this conclusion, another phase 2 study of relapsed or refractory leukemias showed that ruxolitinib was very well tolerated as only 4/38 patients developing ≥grade-3 toxicity (135).

Further ongoing CP-CML trials (NCT02253277, NCT01914484) are seeking to clarify the safety and tolerability profile of nilotinib and ruxolitinib administered in combination, particularly the dose limiting toxicities (DLT) and maximum tolerated dose (MTD). The efficacy of combined therapy is also being considered in a single-arm trial combining ruxolitinib and specific TKI (NCT03610971) by measuring the TFR rate at 12 months. Furthermore, the Southwest Oncology group is conducting a phase 2 study to compare the MR4.5 rate after 12 months of combination therapy with ruxolitinib plus dasatinib or nilotinib versus TKI monotherapy based on local PCR testing (NCT03654768).

Collectively these results and ongoing trials provide new insights into the clinical efficacy of ruxolitinib-based combined intervention in CP-CML, and even other refractory Philadelphia chromosome-positive diseases such as *BCR-ABL*–positive acute lymphoblastic leukemia (ALL) and atypical chronic myeloid leukemia.

## TKI + PPAR-γ Agonists (Pioglitazone)

The combination of the peroxisome proliferator-activated receptor gamma (PPAR-γ) agonist pioglitazone with TKIs in the treatment of CML is now under investigation. Pioglitazone is commonly used to improve glycemic control in people with Type 2 diabetes. PPAR-γ is transcription factor and its activation by pioglitazone serves to increase tissue sensitivity to insulin (136). The finding that pioglitazone induces CML cells to exit their quiescent state *in vitro* and sensitizes them to TKI proposed a strong case for drug repurposing (137). The researchers immediately investigated the possible molecular mechanism of how pioglitazone targets CML LSCs. This revealed that PPAR-γ agonists serve as negative transcriptional regulators of STAT5 and its downstream targets HIF2α and CITED2, which are key guardians of quiescence and stemness in CML LSCs (138).

Imatinib and pioglitazone both down-regulate STAT5 activity, but they work through different pathways. In CML cells, STAT5 is activated upon direct phosphorylation by the BCR-ABL kinase (139). Thus, imatinib inhibits the activation of STAT5 through BCR-ABL phosphorylation, while pioglitazone reduces the expression of STAT5. While imatinib monotherapy is sufficient for clearing more differentiated CML cells, the combination with pioglitazone is more effective since this drives CML LSC to exit quiescence and renders them susceptible to imatinib-induced apoptosis. In a case series, when three CML patients with residual disease were temporarily given pioglitazone under continuous imatinib treatment, they all achieved sustained CMR up to 4.7 years after pioglitazone withdrawal. Furthermore, one patient who stopped imatinib for the last 6 months of his observation period remained in CMR during this period without any treatment. This suggests that TFR may be a generally achievable goal through combination therapies that corrode the cancer stem cell pool (138).

Following promising results of another phase 2 studies (140), prospective randomized studies of pioglitazone synergy with imatinib are currently underway (NCT02767063, NCT02889003).

## Immunological Strategies

As with other malignancies, the immune response against CML can be impaired (141) but is sensitive to immune control. Clinical and experimental studies have documented that the host immune system may effectively suppress or eradicate the quiescent CML stem cells and mediate biological control as a way to succeed in TFR (141–143). Indeed, the only truly curative treatment thus far is allogeneic hematopoietic stem cell transplantation (allo-HSCT) where the anti-leukemia effect of the graft may result from the donor cytotoxic T lymphocytes (CTLs) eliminating residual CML stem cells (144). Over the last decade other novel immunotherapy strategies have included vaccines. BCR-ABL1 from CML patients has been employed as the specific antigen (145) while other leukemia associated antigens (LAAs) have also been used to induce the immune response of T cells against BCR-ABL1 expressing cells (146). The clinical studies in CML using immune strategies including immune checkpoint blockade (ICB) are summarized in **Table 1**.

### Vaccination

#### BCR-ABL1 as a Specific Antigen

As a unique product of gene rearrangement in CML, the BCR-ABL1 oncoprotein can be exploited as an immunogenic target in addition to being a molecular target for inhibitors. While BCR and ABL proteins exist in other normal cells, the peptides that span the junction between BCR and ABL in BCR/ABL oncoproteins are specific to CML cells (145). The breakpoint of *ABL* gene usually occurs at the 5’ region of exon 2 of ABL (a2), but the location of breakpoint in BCR is more variable. In most cases, this occurs between exons b2 and b3 (the b2a2 transcript) or between exons b3 and b4 (the b3a2 transcript) (147). The b3a2 rearrangement is more prevalent, accounting for about 60% of all patients (148). Therefore, the ideal immunogenic peptides of BCR-ABL1 encompass amino acid sequences of the b2a2 or b3a2 breakpoint regions (149). Using this concept, several studies have now explored the therapeutic effect of BCR-ABL1 immunopeptides.

The Evaluation of Peptide Immunisation in CML (EPIC) study recruited 19 CML patients vaccinated with b3a2 peptide. Thirteen patients with cytogenetic response after imatinib treatment showed a late T cell immune response to the BCR-ABL1 peptide, and BCR-ABL1 transcripts decreased by one-log (150). The efficacy of immune peptide cocktails was studied on ten CML patients who expressed b2a2 or co-expressed b2a2/b3a2 BCR-ABL1 subtypes. The BCR-ABL1 mRNA levels were reduced by one-log in three patients, and MMR occurred in another three patients. However, responses were not stable, suggesting this treatment approach only temporarily improves the molecular response in CML patients (151).

In a phase 2 trial (NCT00267085), patients who had previously received imatinib and showed CCyR were vaccinated with CMLVAXB2 or CMLVAXB3 peptides targeting b2a2 and b3a2 BCR-ABL1 subtypes, respectively. At last, three out of ten patients achieved MMR. The interim analysis of another trial of GIMEMA CML Working Party, CML patients with minimal residual disease (MRD) obtained a reduced disease burden after exposed to CMLVAX100 (vaccine derived from BCR-ABL1 b3a2 isoform, adjuvant with molgramostin and QS-21) during imatinib treatment (152). Furthermore, it was reported that the combination of CMLVAX100 and GM-CSF induced a 50% reduction of BCR-ABL1 mRNA levels in patients who were previously exposed to imatinib and/or interferon. This study group also reported one patient with complete molecular responses showing undetectable BCR-ABL1 transcripts in peripheral blood and bone marrow after receiving the b2a2 subtype vaccine (153).

Overall, trials employing vaccines against BCR-ABL1 breakpoints have been shown to reduce residual disease in patients treated with TKI. Further clinical trials remain in progress (NCT00466726, NCT00004052).

#### Leukemia Associated Antigens

Leukemia associated antigens (LAAs) have also been demonstrated to induce immune responses against cells expressing BCR-ABL1 (146). LAAs are over expressed in CML and various LAAs have been identified as potential targets for vaccine synthesis (149, 154). The most promising target so far is the Wilms tumor oncogene (WT1), a zinc finger transcription factor which is often overexpressed in leukemic stem/progenitor cells. Instructively, DMR may be induced when WT1-based immune peptides were used in combined with imatinib (155). A related clinical trial is currently ongoing (NCT00004918).

In conclusion, data from preclinical and clinical reports suggest immune-dependent therapies play an important role in CML treatment but refinements of these approaches remain ongoing.

### Immune Checkpoint Inhibitors

Cancer immunotherapy based on immune checkpoint blockade (ICB) employs monoclonal antibodies against negative immune regulatory checkpoint regulators, such as programmed death 1 (PD-1), programmed death receptor ligand (PD-L1) (156) and cytotoxic T-lymphocyte antigen 4 (CTLA-4). Analyses of the immune activation status in CML suggest ICB approaches are general applicability to this disease.

PD-L1 expressed by tumor cells acts as a co-inhibitory molecule of T cells by binding to PD-1, which is up-regulated on active T cells, leading to T cell exhaustion. Compared with cells from control subjects, cytotoxic T lymphocytes (CTLs, CD3^+^CD8^+^ cells) from CML patients showed higher levels of PD-1, while CML cells expressed higher levels of PD-L1 (157). Moreover, in a mouse model of CML, abrogation of PD-1 could increase overall survival (158, 159). Recently, the correlation between the expression of CD86, the CTLA-4 ligand, and the risk of recurrence after TKI discontinuation was demonstrated. Among 122 patients who had ceased TKIs, those with lower CD86 levels showed a higher (70%) relapse-free survival rate, indicating that CD86 expression may be an early indicator of poor TFR probability (160). Thus, based on these findings, blocking the interaction of PD-1/PD-L1 or employing CTLA-4 blockade may be rational therapeutic approaches for CML. However, the clinical application of this idea has presently been limited.

One completed phase 1 study (NCT02011945) proposed to investigate the safety and efficacy of dasatinib plus nivolumab (PD-1 blockade) in 31 patients with chronic or accelerated CML. However, the study findings are still to be published. Another prospective phase 1 clinical trial (NCT01822509) is presently evaluating the safety and immunologic activity of ipilimumab (161) (CTLA-4 blockade) or nivolumab (162) (PD-1 blockade) for relapsed hematologic malignancies including CML after allo-HSCT.

## Conclusion

Targeted therapy using TKIs is currently the standard treatment for CML patients. Most studies have shown that the general prognosis of patients with CP-CML is excellent as long as they are compliant with the TKI based regimens, monitored regularly and change therapy in time before CML progression. However, TKI therapy is not curative and long-term exposure is associated with considerable patient morbidity as well as burden on health-care systems. TFR has therefore become the significant new goal of CML management but is achievable for only a minority of patients. To completely cure this disease and discontinue TKI, novel complementary interventions need to be explored.

Since there is no information about the rate of successful TFR rate from large randomized trials with different initial treatment regimens, many additional CML trials are currently exploring TFR as the final endpoint such as Italian SUSTRENIM (NCT02602314). This is a prospective phase IV study evaluating both the depth of the molecular response and TFR rates in newly diagnosed CP-CML patients treated with nilotinib or imatinib followed by switch to nilotinib in absence of treatment milestones as per clinical practice. Treatment cessation will be offered after ≥1 year in MR4.0. The purpose of another trial (NCT04043676) is to determine the rate of successful TFR within the first 48 weeks following cessation of treatment in patients who achieved MR4.0 on imatinib and maintained MR4.0 on ponatinib after the switch from imatinib. The phase II DANTE study is going to evaluate the rate of full treatment-free molecular remission in a selected population of CML-CP patients treated with nilotinib at half the standard dose during a consolidation period of 12 months, followed by complete therapy cessation. Furthermore, inspiring new data (163) paves the way for a series of clinical studies that focus on the potential of a combination of asciminib plus catalytic inhibitors to enhance speed of response and contribute to DMR in a greater number of patients relative to single-agent treatments. MDACC investigators are designing a trial to determine the clinical activity of the combination of asciminib and a TKI in CML patients with minimal residual disease (MRD) (NCT04216563). Similarly, MR4.5 rate between asciminib + imatinib and imatinib alone is going to be determined in a phase 2 study from 48 weeks until 96 weeks in CML-CP patients who have been previously treated with imatinib and have not achieved DMR (NCT03578367). The efficacy of treatment with ABL001 in combination with dasatinib and prednisone is also being considered for BP-CML and Ph^+^ ALL patients (NCT03595917). Additionally, the potential of ABL001 with that of bosutinib is being explored now in CML-CP patients previously treated with at least two ATP-binding site TKIs to compare the MMR rate of ABL001 versus bosutinib (NCT03106779). In general, further development of the asciminib-based drug combinations may offer exciting opportunities for more rapid and deeper remissions. In particular there are potential implications for TFR, even preventing the emergence of BCR-ABL1 compound mutations in advanced Ph^+^ leukemias treated with 3G TKI ponatinib, thus further improving long-term outcomes of patients with CML and Ph^+^ ALL. Some of the study questions are attempting to address whether TFR can be accomplished successfully with treatment change or through combination with more powerful agents before treatment discontinuation, which may also increase the pool of eligible patients.

Current thinking suggests TKIs kill dividing CML cells but not quiescent CML stem cell pool, the latter which theoretically need to be eradicated for cure. The discovery of druggable pathways that may be selectively required for CML stem cell survival has led to the repurposing of established non-TKI drugs such as interferon and JAK2 inhibitors as co-treatments with TKIs. In addition, recent advances in understanding CML immunobiology has significantly improved the prospects for developing novel immunotherapeutic strategies such as vaccines and immune checkpoint inhibitors.

Another key goal is to develop an implementable definition of TKI withdrawal for CML patients such that best practice treatment protocols can be applied to clinical practice. Many issues regarding the depth of molecular remission, duration of treatment, predictors, and safety remain open and will continue to be discussed.

The ability to successfully resolve optimized treatments (novel monotherapy or the combination of non-ABL targeted inhibitors/immunotherapy and TKI) and determine the most effective timing for these therapies also remain as future challenges. The key strategies to address these needs are well-designed clinical studies together with accurate prognostic indicators that better predict the persistence of molecular remission of CML patients after TKI withdrawal. Comparisons of quality of life before and after quitting TKI are also important.

In summary, we anticipate that the new therapeutic strategy of TFR will have a significant impact on understanding the determinants of CML treatment. Moreover, while the novel strategies discussed in this review warrant further clinical studies, there is a hope that CML can be completely curable in the foreseeable future.

## Author Contributions

HM and HJ performed a literature search. HM wrote the initial draft, which was revised and amended by XZ, LZ, and HL. All authors contributed to the article and approved the submitted version.

## Conflict of Interest

The authors declare that the research was conducted in the absence of any commercial or financial relationships that could be construed as a potential conflict of interest.
